# An analysis of the relationship between metastases and cachexia in lung cancer patients

**DOI:** 10.1002/cam4.841

**Published:** 2016-08-03

**Authors:** Masatoshi Shiono, Kan Huang, Robert J. Downey, Nikita Consul, Nicolas Villanueva, Kristen Beck, Kathleen Fenn, Donald Dietz, Takuhiro Yamaguchi, Shunsuke Kato, Chaitanya Divgi, Kevin Kalinsky, Ying Wei, Yuan Zhang, Alain C. Borczuk, Akira Inoue, Balazs Halmos, Swarnali Acharyya

**Affiliations:** ^1^ Institute for Cancer GeneticsColumbia UniversityNew YorkNew York; ^2^ Division of Hematology/OncologyDepartment of MedicineColumbia UniversityNew YorkNew York; ^3^ Department of SurgeryMemorial HospitalMemorial Sloan Kettering Cancer CenterNew YorkNew York; ^4^ College of Physicians and SurgeonsColumbia UniversityNew YorkNew York; ^5^ Division of BiostatisticsTohoku University Graduate School of MedicineSendaiJapan; ^6^ Department of Clinical OncologyJuntendo University School of MedicineTokyoJapan; ^7^ Department of RadiologyColumbia University Medical CenterNew YorkNew York; ^8^ Herbert Irving Comprehensive Cancer CenterNew YorkNew York; ^9^ Department of BiostatisticsColumbia UniversityNew YorkNew York; ^10^ Department of Anatomic PathologyWeill Cornell Medical CollegeNew YorkNew York; ^11^ Department of Respiratory MedicineTohoku University HospitalSendaiJapan; ^12^ Department of Palliative MedicineTohoku University HospitalSendaiJapan; ^13^ Montefiore Medical Center/Albert Einstein College of MedicineNew YorkNew York

**Keywords:** Cachexia, KRAS mutation, lung cancer, metastasis

## Abstract

Weight loss and hematogenous metastases are poor prognosis factors in lung cancer patients that can but do not necessarily co‐occur. We retrospectively investigated the clinical association between cachexia, tumor characteristics (such as metastatic burden and mutational status), and treatment in lung cancer patients. The medical records of 394 lung cancer patients from two institutions (Columbia University, USA and Tohoku University, Japan) were reviewed. Information collected included the presence of cachexia, histologic subtype, tumor stage, number of metastases, mutation status, treatment, and survival. Descriptive statistics were performed. Only stage IV patients exhibited >5% weight loss (0.8%, 2.2%, 3.6%, and 5.1%, for stages I to IV;* P* = 0.0001). Patients with metastases developed cachexia more often than patients without metastases independent of treatment (6.0% and 7.1% weight loss in patients with metastases vs. 2.5% and 2.0% in patients without metastases, before [*P* = 0.0001] and after [*P* < 0.0001] treatment, respectively). The change in number of metastatic sites over time correlated with increasing weight loss (5.2%, 10.6%, 13.4%, and 13.4%, for an increase of 0, 1, 2, and ≥3 metastatic sites, from initial diagnosis to the endpoint; *P* < 0.0001). Patients with cachexia had worse survival than patients without cachexia (hazard ratio, 2.94; 95% confidence interval, 2.08–4.16; *P* < 0.0001). Tumors with mutated KRAS were associated with an increased risk of weight loss (11.4% weight loss in patients with mutated KRAS vs. 6.0% in patients with wild‐type KRAS;* P* = 0.0011). Our findings suggest that the capabilities of lung cancer to metastasize and cause cachexia might be linked intrinsically and are independent of treatments administered. KRAS‐mutated tumors were more commonly associated with cachexia.

## Introduction

Cachexia is a complex metabolic syndrome characterized by involuntary loss of muscle mass, with or without loss of fat mass, and is associated with several chronic diseases, including cancer 1. An international definition of the diagnostic criteria for cancer‐related cachexia was published in 2011. This operational definition characterized cancer cachexia as weight loss >5% of total body weight (BW) over the course of 6 months, weight loss >2% in individuals with a body mass index <20 over the course of 6 months, or radiographic evidence of sarcopenia, among other characteristics 2. The diagnostic criteria for cachexia were recently updated, incorporating the two dimensions of weight loss percentage and body mass index 3.

It has been estimated that cachexia affects 80% of patients with advanced cancers 4. Cachexia is most prevalent in patients with pancreatic, gastric, colorectal, lung, and head and neck cancers 5. Cancer cachexia is associated with functional impairment, low tolerance for chemotherapy, fewer symptomatic responses, and increased susceptibility to infections. The negative effects of cachexia often influence treatment by necessitating drug dose reduction, other delays, or discontinuation of treatments 6. Cachexia has a significant effect on patient quality of life (QOL), owing to its association with fatigue and depression.

Epidemiologic studies suggest that the frequency of weight loss in patients with lung cancer is 55%–60%. However, as the onset of weight loss in patients with lung cancer is not as rapid as in patients with pancreatic or gastric cancer, it often remains unrecognized until the end stages of the disease 7, 8. In an analysis of 418 patients with non–small cell lung cancer (NSCLC), the presence of weight loss was thought to be associated with poorer treatment outcomes, attributable to reduced treatment tolerance 9. In a second study, comprising 40 patients with stage III NSCLC, cachexia was associated with lower QOL and shorter survival 10.

Metastasis is the process of systemic dissemination and growth of cancer cells in sites distant from the primary site of disease 11. Since metastases and cachexia may coexist, we sought to investigate the potential link between the development of cachexia and of metastases in a retrospective study of lung cancer patients. We hypothesized that the ability of a cancer to metastasize might be linked to the pathogenesis of cachexia.

## Methods

After approval was obtained from the Institutional Review Boards at Columbia‐Presbyterian Medical Center and Tohoku University, a retrospective review of patients treated for lung cancer at Columbia‐Presbyterian Medical Center and Tohoku University Hospital between May 2004 and May 2014 was performed. Study entry criteria included the pathologic diagnosis of lung cancer, clinical care at one of these institutions for >3 months, and >3 recorded weights. Exclusion criteria included treatment at another institution for >3 months after diagnosis. Demographic and clinical variables were collected (see Data S1), including the presence of clinical comorbidities, such as anorexia and kidney failure, that might also contribute to weight loss.

On the basis of the international definition of cachexia at the time of approval from the Institutional Review Boards, cachexia was defined in this study as >5% weight loss 2. Weights were recorded at defined points during the course of lung cancer diagnosis and treatment, including weight before diagnosis, weight at diagnosis, weight during and after treatment (at approximately 6‐month intervals), and last recorded weight. Baseline pretreatment weight was defined as self‐reported baseline weights or weights from any outpatient visit 1–2 years before the diagnosis of lung cancer. Weight at diagnosis was defined as the weight recorded within 2 weeks of the date of diagnosis of lung cancer. Weight at first treatment was taken as the weight recorded within 3 days of starting the first treatment. To exclude bias resulting from physical differences between patient populations, percentage weight loss instead of absolute weight loss was used for all analysis. Treatment included chemotherapy, radiation therapy, targeted therapy, and surgery. Posttreatment weight was recorded approximately every 6 months. In total, 62 of 394 cases lacked baseline weights; therefore, these patients were not used in analyses of the correlation between pretreatment cachexia and pre‐ and posttreatment metastasis and stage. The weight at the endpoint was defined as the last weight before death, at terminal discharge from inpatient admission, or at the last outpatient visit.

### Statistical analysis

Student's *t* test was used to compare each mean weight loss percentage between characteristics of the two groups, such as presence of pretreatment metastasis, posttreatment metastasis, EGFR mutation, KRAS mutation, and anti‐EGFR tyrosine kinase inhibitor (TKI) therapy. Analysis of variance and Tukey–Kramer's honestly significant difference test were used to compare each mean weight loss percentage among >3 groups, such as histologic subtype (adenocarcinoma, squamous cell carcinoma, small cell carcinoma, other), stage (I, II, III, IV), and chronological metastasis site number change (0, 1, 2, ≥3). Equality of variances of each analysis was confirmed by Bartlett's, Levene's, Brown–Forsythe's, and O'Brien's tests. Kaplan–Meier methods were used to construct survival plots, and the log‐rank test was used to compare the respective groups. Cox proportional hazard regression was used to estimate hazard ratios (HRs) and 95% confidence intervals (CIs). JMP software (version 11.0; SAS Institute, Cary, NC) was used for statistical analyses. In all cases, two‐sided *P*‐values of 0.05 were considered significant.

## Results

### Incidence of cachexia in patients with metastasis pre‐ and posttreatment

The medical records of patients with histologically proven lung cancer treated at Columbia‐Presbyterian Medical Center (*n* = 294) or Tohoku University Hospital (*n* = 100) were reviewed. Patient demographic and clinical characteristics are summarized in Table 1. Given the biological differences between lung cancer subtypes, we asked whether differences exist in cachexia incidence. However, we observed no significant difference in weight loss between patients with the main histopathologic subtypes of adenocarcinoma, squamous cell carcinoma, small cell lung cancer, and others, such as large cell lung cancer (*P* = 0.66; Table 2).

**Table 1:** Patient characteristics (*N* = 394)

Characteristics	Patients
Age, median (range), years	68 (27–96)
Sex	
Male	204 (51.8)
Female	190 (48.2)
Ethnicity	
White	170 (43.1)
Asian	112 (28.4)
Hispanic	61 (15.5)
African American	14 (3.6)
Other	14 (3.6)
Not known	23 (5.8)
Stage	
I	51 (12.9)
II	53 (13.5)
III	103 (26.1)
IV	187 (47.5)
Histologic subtype	
Adenocarcinoma	252 (64.0)
Squamous cell carcinoma	83 (21.1)
Small cell lung cancer	48 (12.2)
Other	11 (2.8)
Chemotherapy	333 (84.5)
First line	333 (84.5)
Second line	142 (36.0)
Third line and beyond	68 (17.3)
Radiation therapy	237 (60.2)
Surgery	144 (36.5)

Data are no. (%), unless otherwise noted.

**Table 2:** Tumor histologic subtype and weight loss (*P* = 0.66)

Histologic Subtype	Patients	Mean weight loss, %
Adenocarcinoma	214	8.7
Squamous cell carcinoma	66	8.1
Small cell lung cancer	41	10.3
Other	10	6.5

We then explored the relationship between the frequency of cachexia in lung cancer patients with different stages of the disease. We found that only patients with stage IV lung cancer (i.e., patients having hematogenous metastases) had a mean weight loss percentage that met the definition of cachexia (Table 3).

**Table 3:** Association between metastasis, stage, and cachexia

Variable	Patients	Mean weight loss, %	*P*
Pretreatment metastasis			0.0001
No	180	2.5	
Yes^a^	151	6.0	
Posttreatment metastasis			<0.0001
No	112	2.0	
Yes	282	7.1	
Stage at diagnosis			0.0001
I	47	0.8	
II	50	2.2	
III	86	3.6	
IV^b^	148	5.1	
Metastasis sites number change^c^			<0.0001
0	169	5.2	
1	67	10.6	
2	62	13.4	
≥3	34	13.4	

a The cases with available baseline body weights were analyzed.

b Three cases were counted as stage III from the above 151 cases, owing to solitary ipsilateral metastasis, according to the UICC 7th edition lung cancer TNM classification and staging system.

c Longitudinal analysis (chronological change from diagnosis to endpoint).

Anticancer therapy such as chemotherapy has systemic effects 12 including decreased oral intake by appetite suppression, nausea, vomiting, and gastrointestinal tract inflammation. Therefore, we analyzed the effect of treatment on weight loss in our patient cohort. We first analyzed mean weight loss in patients with or without cachexia either pre‐ or posttreatment in the context of metastasis. In both pre‐ and posttreatment, the metastatic group had significantly greater weight loss >5% (Table 3).

We next examined whether the number of metastatic sites or the overall tumor volume correlated with the frequency of cachexia. Weight loss was significantly different among the groups according to the existence of hematogenous metastases, both before (*P* = 0.0001) and after (*P* < 0.0001) treatment (Table 3). A longitudinal analysis was performed to assess the chronological change in the number of metastatic sites against the weight loss of each patient; this analysis showed that the weight loss percentage in patients with new metastatic sites was twice that in patients without an increase in metastatic sites (*P* < 0.0001; Table 3).

**Figure 1:**
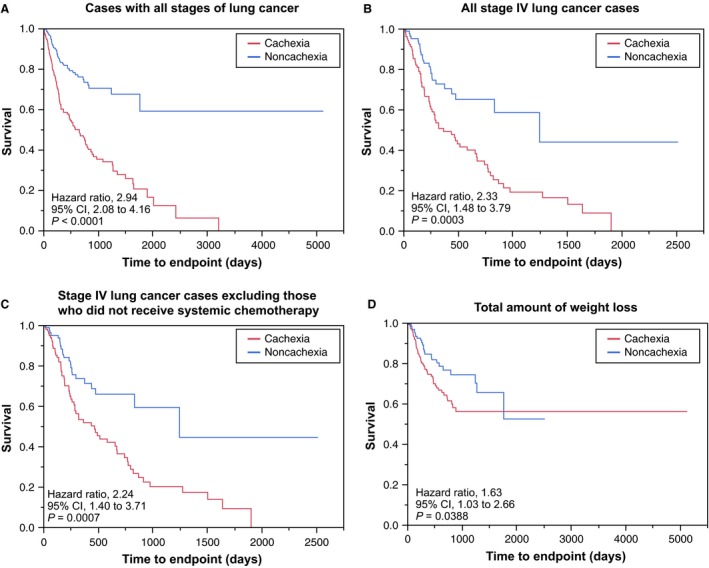
Kaplan–Meier estimates of overall survival among patients with cachexia (defined as >5% weight loss) or without cachexia. (A) Patients with all stages at diagnosis. (B) All stage IV patients. (C) Stage IV patients, excluding those who did not receive systemic chemotherapy and did received only local therapy, such as surgery or radiotherapy, and (D) All patients according to chronological total amount of weight loss.

We performed survival analysis using the Kaplan–Meier method, stratifying patients with and without cachexia. Patients with cachexia before treatment had worse survival (HR, 2.94; 95% CI, 2.08–4.16; *P* < 0.0001; Fig. 1A). In the subgroup of patients with stage IV disease, patients with cachexia at diagnosis had worse survival (HR, 2.33; 95% CI, 1.48–3.79; *P* = 0.0003; Fig. 1B). When patients are deemed cachectic at diagnosis, systemic chemotherapy is often not a viable option. In such instances, poor prognosis could be attributed to not only cachexia but also to lack of therapy administration. We considered such a possibility and carried out an analysis that excluded patients who did not receive systemic chemotherapy (i.e., who received only locally ablative therapy, such as surgery or radiotherapy). We observed a significantly poorer prognosis in the cachexia group (HR, 2.24; 95% CI, 1.40–3.71; *P* = 0.0007; Fig. 1C). Finally, in posttreatment setting, a longitudinal analysis using the total amount of weight loss during treatment, from the time of diagnosis, showed that patients with progressive cachexia had significantly worse survival (HR, 1.63; 95% CI, 1.03–2.66; *P* = 0.0388; Fig. 1D).

### Mutation status in tumors and cachexia incidence

In the era of genomic and personalized medicine, lung cancer treatment is commonly guided by molecular testing of key driver mutations such as KRAS and EGFR 13, 14. KRAS and EGFR represent two most commonly mutated oncogenes in lung cancer with distinct biology. KRAS and EGFR encode for a G‐protein and transmembrane tyrosine kinase, respectively, and both have a critical role in proliferation and cell survival. Therefore, we examined whether the risk of cachexia was associated with the mutation status of these genes. Among several other characteristics that were explored (Table 4), the presence of mutated KRAS in tumors was associated with a doubled risk of weight loss (*P* = 0.0011; Table 4); EGFR mutation status was not associated with an increased risk of weight loss (*P* = 0.6032; Table 4). Because TKIs, such as gefitinib and erlotinib, significantly improve the clinical outcome of EGFR‐mutant lung cancers, we also analyzed cachexia risk in patients with EGFR mutation by treatment with anti‐EGFR TKIs. However, no statistically significant difference in weight loss percentage was found by TKI treatment status (*P* = 0.3036; Table 4). Therefore, these results suggest that KRAS mutation status of tumors, and therefore, their intrinsic biological characteristics could be linked to cachexia development.

**Table 4:** Association between tumor mutation analysis and weight loss

Variable	Patients	Mean weight loss, %	*P*
EGFR mutation			0.6032
Yes	55	7.7	
No	156	8.5	
KRAS mutation			0.0011
Yes	52	11.4	
No	117	6.0	
Anti‐EGFR TKI therapy in the EGFR‐mutant group			0.3036
Yes	22	9.4	
No	33	6.6	

## Discussion

The incidence of weight loss in patients with lung cancer is estimated to be 55%–60%, but the associations between the histologic and clinical characteristics of the cancer and the likelihood of cachexia are poorly characterized. The present retrospective analysis of clinical data from lung cancer patients has four main findings. First, patients with metastatic lung cancer had higher rates of cachexia, both before and after treatment, compared to patients without metastatic disease. That cachexia is seen in the pretreatment or treatment‐naive setting argues against the hypothesis that cancer‐associated cachexia is primarily caused by treatment (e.g., chemotherapy‐induced gastrointestinal disorders or radiation sickness) and suggests that it is likely an inherent characteristic of the tumor. Second, within the cohort of patients with metastases, a greater burden of metastatic disease (as measured by the number of metastatic sites) correlated with a higher risk of cachexia. Third, the presence of cachexia predicted poorer survival, independent of treatment. Fourth and finally, the presence of mutated KRAS in tumors correlated with likelihood of cachexia.

KRAS‐ and EGFR‐mutant lung cancers display different biological characteristics, are clinically distinct, and are treated differently 15. KRAS‐mutant tumors account for approximately 25% of cases of NSCLC, whereas EGFR‐mutant tumors account for 10%–35%. KRAS mutation status has predictive value for colorectal cancer patients receiving anti‐EGFR antibody treatment, but its diagnostic significance for lung cancer patients is less clear 16, 17. Generally, EGFR and KRAS mutations appear to be mutually exclusive 18. Our study showed that the presence of a KRAS mutation in the tumor correlated with the development of cachexia, whereas EGFR mutation was not significantly correlated with cachexia. Interestingly, the mean weight loss did not significantly differ for patients with EGFR mutations by anti‐EGFR TKI therapy status, despite it being an effective targeted therapy for lung cancer 19, 20. Therefore, the correlation between tumor‐associated mutations and cachexia must be related to the inherent mutation and its associated aggressiveness, rather than to the associated treatment. In line with these clinical findings, mouse models bearing lung, colon, or pancreatic cancers harboring KRAS mutations have been found to exhibit features of cachexia 21, 22, 23, 24. As KRAS mutation is one of the key oncogene drivers linked to aggressiveness in various human malignancies—including lung, colorectal, and pancreas cancers 25, 26—it is plausible that mutated KRAS drives both metastasis and cachexia and, consequently, correlates with poor prognosis. However, our results, although suggestive of such an association, require further validation.

ALK rearrangement, MET amplification, BRAF mutation which account for 3–7%, 2–4%, and 1–3%, respectively, represent less frequent alterations in lung cancer 27. Future studies are needed to identify the association of other genomic alterations besides KRAS and EGFR and cachexia development. Interestingly, an experimental mouse model of human anaplastic thyroid cancer developed from injection of a cell line harboring BRAF^V600E^ and TP53^R248G^ mutations developed both metastasis and cachexia. Future studies are warranted to identify whether a similar link between BRAF^V600E^ and cachexia exists in patients.

It can be envisioned that cancer progression impacts skeletal muscles by diverse mechanisms. In experimental models, Waning et al. showed that the bone metastatic tumors induce osteolysis and cause TGF beta release into the circulation 28, 29. TGF beta induces intracellular calcium leak and skeletal muscle weakness. Other soluble factors such as tumor‐derived parathyroid hormone‐related protein (PTHrP) in a lung cancer model mediates energy wasting in adipose tissue and affects skeletal muscle mass and strength 30. Release of extracellular vesicles or exosomes in cancer is yet another potential mechanism which could link systemic effects of metastasis with skeletal muscle wasting. Exosome release and function has been implicated in the premetastatic and metastatic states of cancer progression 31, 32, 33. He et al. 34 showed how tumor‐derived microvesicles could impact distant skeletal muscle by inducing apoptosis of skeletal muscle cells and muscle mass loss. Future experimental studies are needed to mechanistically define the contribution of metastasis to cachexia development.

In our present retrospective study, there are limitations that need to be considered. First, important parameters such as muscle composition and function could not be determined. Impaired muscle function has been recently linked to excess TGF beta released from bone metastasis in mouse models 28, suggesting that these might be relevant parameters to test in future prospective trials linking metastasis and cachexia. Second, BW measurement may not accurately reflect body composition. Intravenous infusions can lead to increased BW (volume) and even fluid overload 35. Similarly, lung cancer patients may develop malignant pleural or peritoneal effusions, leading to an increase in overall BW, even if there is loss of muscle and fat stores. Hypoalbuminemia can lead to third spacing of fluid, owing to a decrease in oncotic pressure in the face of maintained vascular hydrostatic pressure, and can raise BW. However, despite these potential confounders, our study shows a consistent strong relationship between weight loss and metastasis. Two other parameters—obesity masking loss of muscle mass and cachexia symptoms preceding measured weight loss—could also lead to underestimation of the influence of cachexia on QOL and survival among cancer patients 6. Such limitations could not be addressed in this retrospective study but are being pursued in our prospective studies on metastasis and cachexia using longitudinal image analysis, following published studies that used imaging modalities to characterize cachexia 36, 37, 38.

In summary, our retrospective clinical analysis of patients with lung cancer suggests that there is a link between metastasis and cachexia that is associated with the inherent tumor characteristics, rather than with treatment. Our broad research goal is to develop a comprehensive diagnostic/predictive platform that integrates molecular and clinical features of lung cancer with metastatic ability and risk of cancer cachexia. The present findings lay the groundwork for ongoing investigations and provide the rationale for future prospective and experimental studies for further understanding of and ability to treat lung cancer cachexia.

## Conflict of Interest

No conflict of interest from any authors.

## Supporting information


**Data S1.** Notes for data collection.
