# Supplementary material for: An analysis of the relationship between metastases and cachexia in lung cancer patients
Source: Cancer Med. 2016 Aug 3;5(9):2641–8. doi: 10.1002/cam4.841 (PMC5055184; doi:10.1002/cam4.841)
Supplement: Supplementary file 1 — Data S1. Notes for data collection. [file CAM4-5-2641-s001.pdf]

Supplemental Text.

Notes for Data Collection

Date of first treatment: Treatment includes chemotherapy, radiation therapy, or surgery (has to be cancer treatment related), whichever happened first.

Date of Diagnosis: Date from the pathology report of initial definitive diagnosis.

Stage: The first stage scans upon diagnosis by PET/CT or CT chest/abdomen/pelvis and brain imaging.

Metastatic sites at diagnosis: The first staging scan upon diagnosis. Only evident metastases from imaging studies or pathology must be recorded. Nonspecific findings, which may or may not be cancer, must be excluded. Contralateral or ipsilateral lung and pleural disease must be recorded separately from parenchymal disease.

Metastatic sites at endpoint: Same as metastatic sites at diagnosis.

For the above two, abbreviations should be standardized as follows:

Met sites	Brain	contralateral lung	ipsilateral lung	Pleural Disease	Osseous dz	Adrenal Dx	Liver	Pleural Effusion	Other sites
Abbreviations	CNS	C-Lung	I-Lung	P-D	Bone	Adr	Liv	P-E	text

Mutation status: Whatever is available from path report-EGFR/ALK/K-RAS/B-Raf/Met.

Histology:

Histology	Adenocarcinoma	Squamous Cell ca	Small cell Lung ca	neuroendocrine	Large cell Lung ca
Abbreviations	Ade	SCC	SCLC	NE	LCLC

Sarcomatoid lung cancer	poor-differentiated lung Ca	Carcinoma of unknown primary origin	Others rare histology	Not known<br>Or Path not available

Sar	PD	CUP	Text	DK

Treatment: Regimen and starting/ending date of regimen must be recorded.

In general, use the three letters of generic names chemotherapy drugs as follows:

Generic name	Cisplatin	Etoposide	Pemetrexed	Paclitaxel	Carboplatin	Gemcitabine	Docetaxel
Other names	CDDP	VP-16	Alimta	Taxol	Paraplatin	Gemzar	Taxotere
abbreviations	Cis	Eto	Pem	Pac	Car	Gem	Doc

Generic name	Vinorelbine	Bevacizumab	Cetuximab	Topotecan	Erlotinib	Irinotecan	Others
Other names	Navelbine	Avastin	Erbitux	Hycamtin	Tarceva	Camptosar, CPT-11	
abbreviations	Vino (4 letters)	Bev	Cet	Top	Erl	Iri	Text

Surgery: Y/N (Yes/No) and date should be recorded if it was related to cancer treatment or palliation.

Radiation: Y/N.

Baseline weight (BW): The weight from any outpatient clinic visits 1-2 year prior to diagnosis of lung cancer. If there is a fluctuation of weight in the window, then take the average of 4 weights approximately close to 2 year, 1.7 year, 1.4 year and 1.1 year respectively prior to lung cancer diagnosis. If there was no aforementioned information, use the summed weight of "recorded initial clinic visit weight plus the patient self-reported weight loss."

Weight at diagnosis: The weight recorded closest to time of diagnosis within 2 weeks is acceptable.

Weight of first treatment (WOFT): The weight recorded within 3 days of starting first treatment. Same as date of first treatment, treatment should include chemotherapy, radiation therapy, or surgery.

Weight loss pretreatment: BW minus WOFT, can be negative if any weight gain.

Percentage of weight loss pretreatment: (BW minus WOFT)/BW, also can be negative if any weight gain.

Post-treatment weight (PTW):

1. Last weight recorded at expiration, terminal discharge, last recorded visit at outpatient visit.

2. For stage I - III lung cancer, use the weight recorded at 3 month after last-treatment (chemotherapy, radiation therapy, surgery) as endpoint if there was no evidence of recurrence for last 2 years by surveillance image. If there was a recurrence, then use last weight recorded at expiration, terminal discharge, last recorded visit.

Post-treatment weight loss in kg: WOFT minus PTW, can be a negative if any weight gain.

Post-treatment weight loss category:

Percentage of weight loss post treatment	⩾ 10%	⩾ 5% and <10%	< 5% or no change	Any weight gain
Weight loss category	3	2	1	0
Classification at analysis	Severe	moderate	mild	None

% of weight loss post Treatment: (WOFT minus Post treatment weight)/WOFT

Total amount of weight loss: Pretreatment weight loss + post treatment weight loss.

Survival days: Date of death minus date of cancer diagnosis, N/A if patient is alive.

List of significant medical comorbidities: Chronic obstructive pulmonary disease (COPD), Depression, Anorexia, Cirrhosis, Another cancer besides lung cancer, Heart failure (on treatment or EF <40%), Kidney failure on replacement therapy, Thyroid disease (on treatment), Diabetes (on treatment other than metformin), HIV/AIDS, History of any transplantation in the past, Dementia, Crohn’ s disease or Ulcerative colitis, Celiac disease, Stomach ulcer, Pancreatitis, Esophageal disease, Addison’s disease, History of digestive tract surgery, Stroke or Cerebral

vascular disease, Paralysis by all causes, Schizophrenia or severe mental illness, Venous or arterial thromboembolism

Performance status (PS): Eastern Cooperative Oncology Group PS at diagnosis.

Smoking status: Record number of pack year.

Albumin/WBC/Hemoglobin/Platelet at diagnosis or endpoint: Those at same definition of the weight at baseline or endpoint treatment.

Medications: All medications taken at any given time during the course of treating cancer, which can influence the weight such as steroid, synthroid, diuretics.
